# Bioaccessibility of antimony and other trace elements from lead shot pellets in a simulated avian gizzard environment

**DOI:** 10.1371/journal.pone.0229037

**Published:** 2020-02-11

**Authors:** Amanda D. French, Katherine Shaw, Melanie Barnes, Jaclyn E. Cañas-Carrell, Warren C. Conway, David M. Klein

**Affiliations:** 1 Department of Environmental Toxicology, The Institute of Environmental and Human Health, Texas Tech University, Lubbock, Texas, United States of America; 2 Department of Geosciences, Texas Tech University, Lubbock, Texas, United States of America; 3 Department of Natural Resources Management, Texas Tech University, Lubbock, Texas, United States of America; Chinese Academy of Sciences, CHINA

## Abstract

Many studies have used grit (in the form of lead (Pb) pellets) presence in avian gizzards as an indicator of Pb shot exposure. However, due to nearly complete pellet absorption in gizzards or rapid passage of pellets, the absence of Pb shot presence in a gizzard does not confirm lack of Pb shot exposure. This study provides the basis for an additional technique to identify if elevated tissue Pb concentration is due to Pb shot exposure. Bioaccessibility of Pb and trace elements (Sb, As, and Sn) present in Pb shot were quantified to determine if any of these elements would be useful as a secondary marker of Pb shot exposure. An avian physiologically based extraction test (PBET) was used to determine pellet dissolution rate and bioaccessible concentrations of Pb, Sb, As, and Sn in a simulated gizzard environment. Of the three trace elements, only Sb concentrations (44–302 μg/mL) extracted into the gizzard solution were greater than environmental background levels (US soil average 0.48 μg/g); thus, no natural source likely provides this amount of Sb. Therefore, there is evidence that Sb can be extracted from Pb shot in bird gizzards at detectable concentrations (above natural background). While further studies are needed to delineate the mechanisms of absorption and distribution, this study lends credence to the hypothesis that Sb may be a useful marker of Pb shot exposure in biological tissues, particularly when Pb pellets are not present nor observed in avian tissues.

## Introduction

Lead (Pb) shot is known to be a major route of Pb exposure in birds and continues to be a global concern [1–15]. Exposure can happen through four major pathways: direct consumption of Pb shot pellets, indirectly through ingestion of soil or food contaminated with Pb shot, absorption from embedded pellets in wounded animals, and ingestion of dead or living animals that have been shot with Pb shot (mainly affecting scavenging species) [8]. These pathways can be deleterious and significant sources of Pb exposure, but precisely identifying Pb shot (rather than other environmental or anthropogenic sources of Pb) as the source of Pb exposure remains challenging.

Despite the Pb shot ban in place for waterfowl hunting in many countries, an estimated 1,900–2,400 metric tons of Pb shot are deposited on publicly owned lands across the US each year from mourning dove (*Zenaida macroura*) hunting alone [7] and an estimated 18,000–21,000 tons of Pb (ca. 200 billion individual pellets) are used for hunting annually in Europe [11,16]. Deposition of Pb shot throughout the environment is not uniform, where areas hunted annually will have a greater pellet density in the environment than those less frequented by hunters. For example, pellet deposition has been estimated to be as high as 167,593 pellets ha^-1^ in dove hunting fields (New Mexico, USA) [17], 2.8 million pellets ha^-^1 in wetlands (Spain) [9], and Mateo (2009) reports shot densities as high as 3.99 million pellets ha^-^1 in the upper 30 cm of wetland sediments in southern Europe [18]. These high deposition areas can have direct impacts on soil, plants, waterfowl [4,9,19], passerine species [2,20,21] and many other birds, both game and non-game species [8,22–25]. As such, Pb shot deposition remains a relevant, and widespread issue [7,11,26,27].

Lead shot typically contains 0.5–6.5% Sb, 0.1–0.2% As, and ~0.1% Sn, depending on the manufacturer [28]. These additives produce a more desirable end product, where Sb increases pellet hardness, As facilitates sphere formation, and Sn increases malleability and reduces fragmentation [28,29]. If these elements are present at concentrations above background levels, they may be useful as tracers or secondary markers for identifying sources of Pb from pellets specifically in both environmental media and biological tissues. Based on the deposition of Pb shot in the environment and assuming an average Sb concentration in Pb shot of 3%, approximately 57–72 metric tons of Sb is deposited into the environment each year from Pb shot pellets alone. Background soil-Sb concentrations range from < 1.0 to 8.8 μg/g with an average of 0.48 μg/g in the United States [30], but few have reported Sb concentrations in avian tissues, and no estimates of toxicological thresholds currently exist for Sb in birds [31]. As such, compared to natural concentrations and availability of Sb, Pb shot is likely a major anthropogenic source of Sb exposure in birds. Lead shot remains in the environment for up to 300 years [32,33] often found in the upper 3 cm of soil [20], leaving pellets readily accessible to birds and mammals that feed in the soil or on plants and invertebrates that uptake trace metals and Pb from soil contaminated with Pb shot.

Lead pellet exposure in birds has been studied for decades, although source identification of Pb shot in biological tissues remains complicated for a variety of reasons [25,34–37]. The primary complication with Pb source identification is that an individual organism may be exposed to multiple sources of both natural and anthropogenic Pb. While Pb isotope ratios have been frequently used to identify Pb sources, precision of those isotopic techniques declines when multiple Pb sources are mixed, leading to averaging, or less precise isotope ratios that no longer indicate a single source. Another issue in using isotope ratios is the growing use of recycled Pb. When multiple Pb sources are recycled to form a different product, a less distinctive isotopic ratio results and source determination becomes much more difficult than using non-mixed Pb isotopes alone [38]. The aim of this study is to provide another method, to use in conjunction with Pb isotope ratios and aid in distinguishing Pb shot exposure.

Both herbivorous and granivorous birds require grit to aid in digestion, as their food requires additional mechanical processing prior to digestion [39]. Such birds, most notably within Galliformes and Anseriformes, typically use grit in conjunction with a large muscular gizzard. In contrast, many insectivorous birds require less, or no grit [39] and typically have a smaller gizzard. Furthermore, many species switch diets seasonally and grit requirements vary accordingly based upon nutritional demands and timing within their annual cycle [40]. Typically, birds that purposefully consume grit have greater exposure risk to Pb shot, as pellet size and shape are consistent with the size and shape of naturally occurring grit [20,23,41]. As such, retention rate of Pb shot pellets varies dramatically among species [41,42]. For example, Northern bobwhites (*Colinus virginianus*) exposed to Pb shot pellets absorbed or excreted pellets within 14 days of exposure [43] and brown-headed cowbirds (*Molothrus ater*) dosed with Pb shot excreted pellets within 24 hours of dosing, while those that did not excrete pellets died [44]. Further, house sparrows (*Passer domesticus*) retained grit for 5 days [45] and Pekin ducks (*Anas platyrhynchos forma domestica*) lost nearly 50% of their body mass during a 4-week experiment [46]. Thus, Pb shot absorption and retention is quite variable, and influenced by species, the type and amount of grit ingested [47], diet and nutritional demands, sex, season, and particle size [42].

Many studies simply use presence/absence analyses of Pb pellets in the gizzard or gastrointestinal (GI) tract to identify Pb shot exposure [3,48–51], but some species may excrete grit rapidly, such that it might not be detected, even if previously consumed. Alternatively, individuals that lack Pb pellets in gizzards may have already broken down and absorbed those pellets, where Pb pellet absence does not confirm lack of Pb shot exposure. Despite these potentially overlooked issues in gizzard analyses, long-term data sets and monitoring may indicate temporal changes in pellet availability on the landscape [52]. Species that consume softer foods, such as American woodcock (*Scolopax minor*), are thought to be exposed to Pb shot either through direct pellet or soil consumption [25]. However, there are no reports of Pb shot pellets in woodcock gizzards [25,53,54]. Strom et al. (2009) and Scheuhammer et al. (2003) suggest that the absence of Pb pellets in woodcock is due to the lack of a large muscular gizzard, which allows relatively rapid pellet passage and excretion, minimizing total exposure time. Similarly, some songbirds excrete Pb shot pellets within 24 hours, but still exhibit symptoms of lead toxicity, where pellet Pb erosion may reach 42 mg/kg [44]. This suggests that even limited temporal exposure to Pb shot pellets may elevate tissue Pb concentrations enough to potentially cause health issues, even for species that do not intentionally target Pb pellet for consumption as grit or food [44].

Bioaccessibility, or the total fraction available for absorption, of Pb from Pb shot pellets has been investigated previously [47,55]. However, these studies do not evaluate bioaccessibility of other elements commonly present in Pb shot (Sb, Sn, or As) [28], presumably because of the relatively low concentration of these elements in comparison to Pb. Due to the challenges associated with definitively identifying Pb sources, quantifying the bioaccessibility of these trace-elements, specifically Sb, would be a valuable step in determining the feasibility of using Sb in future avian Pb exposure studies as a conclusive marker of Pb shot exposure. Therefore, this study determined bioaccessible concentrations of Pb, Sb, As, and Sn from Pb shot pellets to verify that these elements are extracted into a (simulated) gizzard solution and therefore available for absorption into avian tissues.

## Methods

### Simulated gizzard intestinal solutions

Similar to Martinez-Haro et al. (2009), avian gizzard digestive juices were simulated by preparing a solution of 1 M NaCl (Sigma-Aldrich, St. Louis, MO) containing 10 g L^-1^ of pepsin (Sigma-Aldrich, St. Louis, MO) and adjusting to pH 2.0 using hydrochloric acid (HCl; Fisher Scientific, Hampton, NH). The pH of the gizzard is typically more acidic (2.0–3.2) [55] than the intestines (5.2–7.2) [56,57], varying with the amount of food present in the GI tract [56]. Following previous studies [46,55,57,58], a pH of 2.0 was used to prepare the simulated gizzard solution.

### Dissolution study

Based on preliminary data [31], only Pb shot was used for the dissolution study, as steel shot was not found to contain Pb, Sb, As, or Sn. Three brands of Pb shot from three production years [Federal (2011), Winchester (1965 and 2017), and Remington (2017)] were used to determine variation in trace-metal concentrations among brands. All pellets used for each brand-year combination were removed from the same shotgun shell to minimize any variation among shells. For each brand, three replicate samples were prepared by pipetting 12 mL of gizzard solution into separate 50 mL centrifuge tubes. Seven replicate samples were used for the Federal (2011) shot as a preliminary study prior to using other pellet brands. The sample replicate number was decreased to three for Winchester and Remington shot due to the large number of samples produced. Mean pellet mass for each brand was 66.9 ± 1.5 mg (*n* = 3) for 1965 Winchester pellets, 70.6 ± 4.5 mg (*n* = 3) for 2017 Winchester pellets, 80.2 ± 2.3 mg (*n* = 3) for 2017 Remington pellets, and 78.2 ± 12.7 mg (*n* = 7) for 2011 Federal pellets. Each sample tube contained one pellet to determine the dissolution rate of a single pellet from each brand.

All samples were placed in a MaxQ 4000 incubated benchtop orbital shaker (Thermo Scientific, Waltham, MA) at 42 ± 2°C and shaken at 350 rpm. Rotation speed and temperature were chosen based on biologically relevant values and previous studies [47]. Every six hours, 5 mL of solution was removed and replenished using fresh gizzard solution to replicate the biological solutions moving from the gizzard to the intestines (where absorption occurs). Each volume of solution that was removed was stored in a 15 mL sample tube for analysis via inductively coupled plasma–mass spectrometry (ICP-MS). Prior to gizzard solution replacement, each pellet was carefully removed using clean, disposable antistatic microspatulas (VWR, Radnor, PA) to measure the pellet mass at each 6 hour sampling period. This process was continued until each pellet replicate was completely dissolved within the simulated gizzard solution. Pellet dissolution rate was calculated using Eq 1:

$$dissolution\;rate=\frac{\left(P_{initial}-P_{final}\right)}{total\;time\;(hrs)}$$ (1)

where P_initial_ is the initial pellet mass (mg), P_final_ is the pellet mass (mg) at each time point, and total time is the time the pellet has been in solution (in hours). This dissolution rate estimates temporal stability in pellet mass loss, and aids in predicting complete dissolution time based upon pellet mass.

### Elemental Analysis

Concentrations of As, Sn, Sb, and Pb were quantified using a Perkin Elmer Elan DRC-e ICP-MS. A calibration curve ranging from 0.0001–0.50 μg/g was prepared using SPEX CertiPrep CL-CAL-2 standard. Samples collected at each time point were diluted 1:100 using a stock solution of 2% HNO_3_ and 1% HCl. Each sample was spiked with 0.10 μg/g of an internal standard (Inorganic Ventures VAR-IS-1) containing Bi, In, Li, Sc, Th, and Y. Continuing calibration verification (CCV) standards and reagent blanks (containing 2% HNO_3_, 1% HCl and internal standard) were analyzed at least every 18 samples to ensure calibration concentrations remained stable (<15% variation). Arsenic was analyzed using the Dynamic Reaction Cell^TM^ (DRC^TM^) to react As with oxygen producing AsO^+^. This shifts ^75^As away from interferences (such as ^40^Ar^35^Cl^+^) to decrease the detection limit for As.

The detection limit (DL) for each element was as follows: As = 0.0003 μg/g, Sn = 0.0006 μg/g, Sb = 0.0001 μg/g, and Pb = 0.0009 μg/g. Lead was determined semi-quantitatively since concentrations were greater than the maximum point on the calibration curve (0.50 μg/g). This was considered acceptable for this study as the focus was on the trace metal(loid)s (As, Sn, and Sb). All CCV samples varied by < 10% throughout the analysis. Average method blanks (consisting of only gizzard solution) had < 0.0005 μg/g As, Sn, and Sb. The method blanks for Pb were greater (0.0011 ± 0.0016 μg/g); but were considered acceptable since Pb concentrations in the samples were much greater than this.

### Bioaccessibility calculations

Pellet mass loss was used to determine the bioaccessible fraction (% BA) of As, Sn, Sb, and Pb. Total concentrations for each element were calculated following Eq 2:

$$Total=C_{T1}+\sum \;\left|(0.58\;x\;C_{Ti})-C_{j}\right|$$ (2)

where C_T1_ is the concentration at the first time point, 0.58 is the portion of the concentration contributed from the previous time point (5 mL removed for analysis, 7 mL remaining divided by 12 mL total volume), C_Ti_ is the concentration found at a given time point, and C_j_ is the concentration found at the next time point analyzed. For example, a concentration of 2.4 μg Sb/mL was determined at the 6-hour time point and 2.7 μg Sb/mL was determined at the 12-hour time point. To calculate the total concentration following Eq 2: 2.4 + |(0.58x2.4)-2.7| resulting in a final concentration of 3.7 μg Sb/mL gizzard solution. The total concentration was calculated for As, Sn, Sb and Pb for all time points analyzed for each shot type and this value was used as the total concentration in Eq 3 and the extracted concentration is the amount of As, Sn, Sb, or Pb present in the gizzard solution at each time.

$$\%BA=\frac{extracted\;[]}{total\;[]}x\;100$$ (3)

It is important to note that not all time points were analyzed for trace element concentrations. This was simply due to the large number of samples generated. Therefore, samples were analyzed every 6-hours for the first 36-hours and then every 12-hours for the remainder of the study.

## Results

### Dissolution study

**Fig 1:**
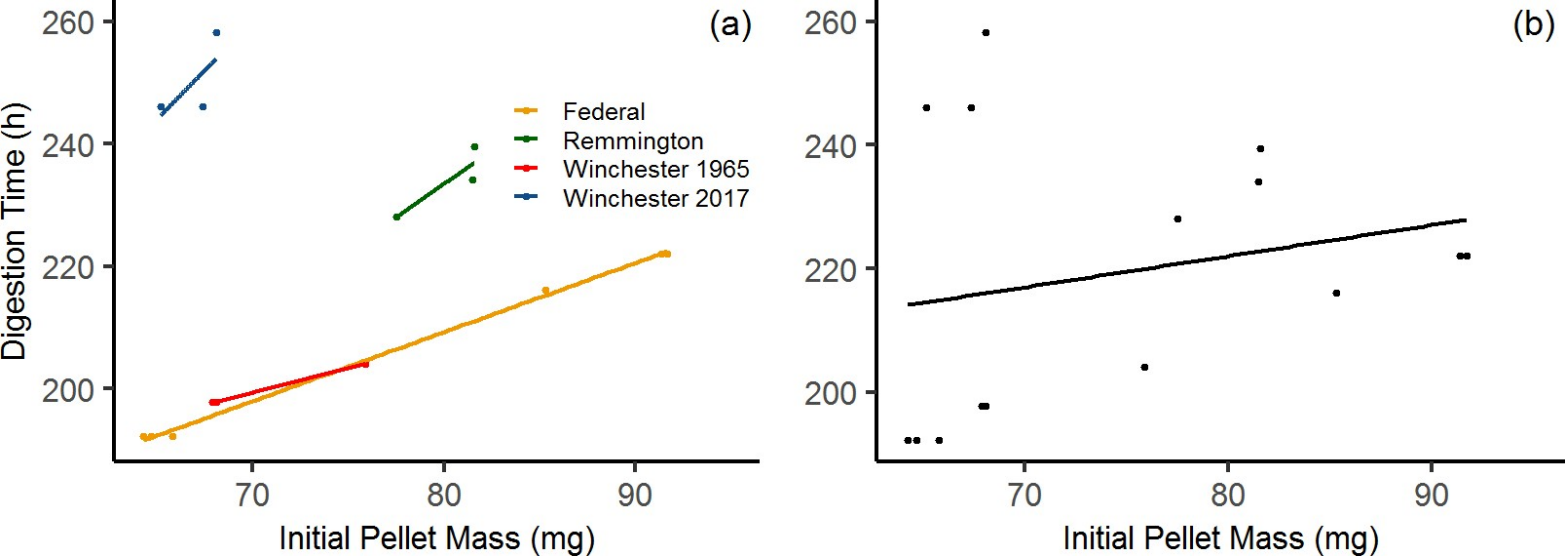
Individual and cumulative trends in total dissolution time compared to initial pellet mass. **(a)** Individual brand trends, where positive correlation is strongest in the Federal (2011) brand, likely due to larger sample size (*n* = 6). Correlation coefficients for each brand are as follows: Winchester (1965): r^2^ = 0.472; Winchester (2017): r^2^ = 0.986; Remington (2017): r^2^ = 0.793; Federal (2011): r^2^ = 0.998; **(b)** Cumulative trends (*n* = 16). Correlation coefficient for the overall trend is: r^2^ = 0.049.

The average time to complete dissolution was 200 ± 3.6 h for the 1965 Winchester pellets (*n* = 3), 250 ± 7.0 h for the 2017 Winchester pellets (*n* = 3), 234 ± 5.7 h for the 2017 Remington pellets (*n* = 3), and 206 ± 15 h for the 2011 Federal pellets (*n* = 6; S1 Fig). The number of Federal pellets decreased by one because one pellet was lost early on in the experiment. Total digestion time was positively correlated with initial pellet mass for each brand (Fig 1A), but correlation strength declined when all brands were examined simultaneously (Fig 1B). The most important factor explaining total digestion time was initial pellet mass (*P* < 0.01), where those with the greatest digestion times had greater As concentrations and lower Sb concentrations, while Sn concentrations were variable over the digestion time.

**Fig 2:**
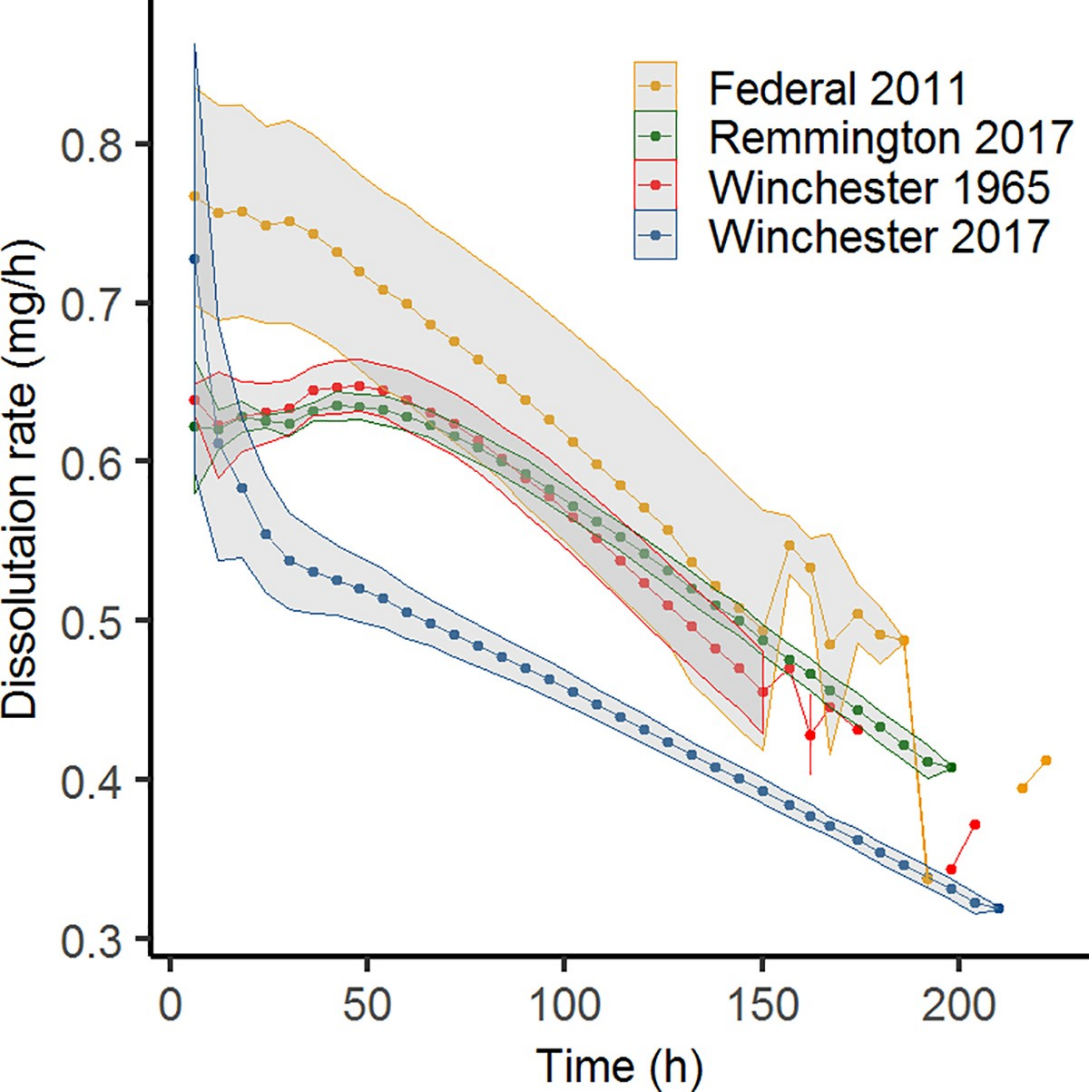
Pellet dissolution rate of the four brand-year types of Pb shot. Shading represents the standard deviation of the replicate samples (*n* = 6 for Federal shot, *n* = 3 for all other shot types).

**Fig 3:**
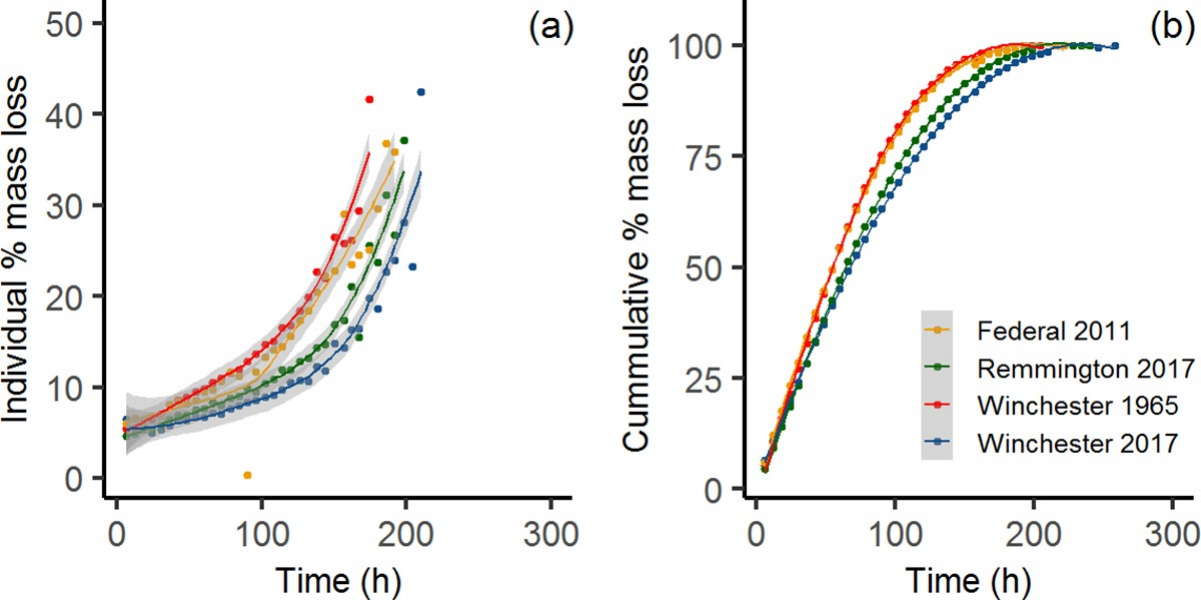
Individual and cumulative pellet mass loss of the four brand-year types of Pb shot. **(a)** Individual mass loss (%) and **(b)** cumulative mass loss (%). Solid line represents the line of best fit along and shaded areas are the 95% confidence interval (*n* = 6 for Federal shot, *n* = 3 for all other shot types).

The average dissolution rate (mg/hr) for each brand ranged between 0.45–0.60 mg/hr [1965 Winchester pellets 0.45 ± 0.09 mg/hr (*n* = 3), 2017 Winchester pellets 0.55 ± 0.09 mg/hr (*n* = 3), 2017 Remington pellets 0.55 ± 0.08 mg/hr (*n* = 3), and 2011 Federal pellets 0.60 ± 0.12 mg/hr (*n* = 6)]. For all four sample types, dissolution rate declined over time (Fig 2), but percent mass loss at each interval increased. As the total mass remaining declined, the proportion of mass lost was greater at each sampling interval (Fig 3A). Maximum percent mass loss at any given time point for each brand were as follows: Winchester (1965) 60%; Winchester (2017) 41.7%; Remington (2017) 38.5%; and Federal (2011) 45.5%.

### Concentration analysis

**Fig 4:**
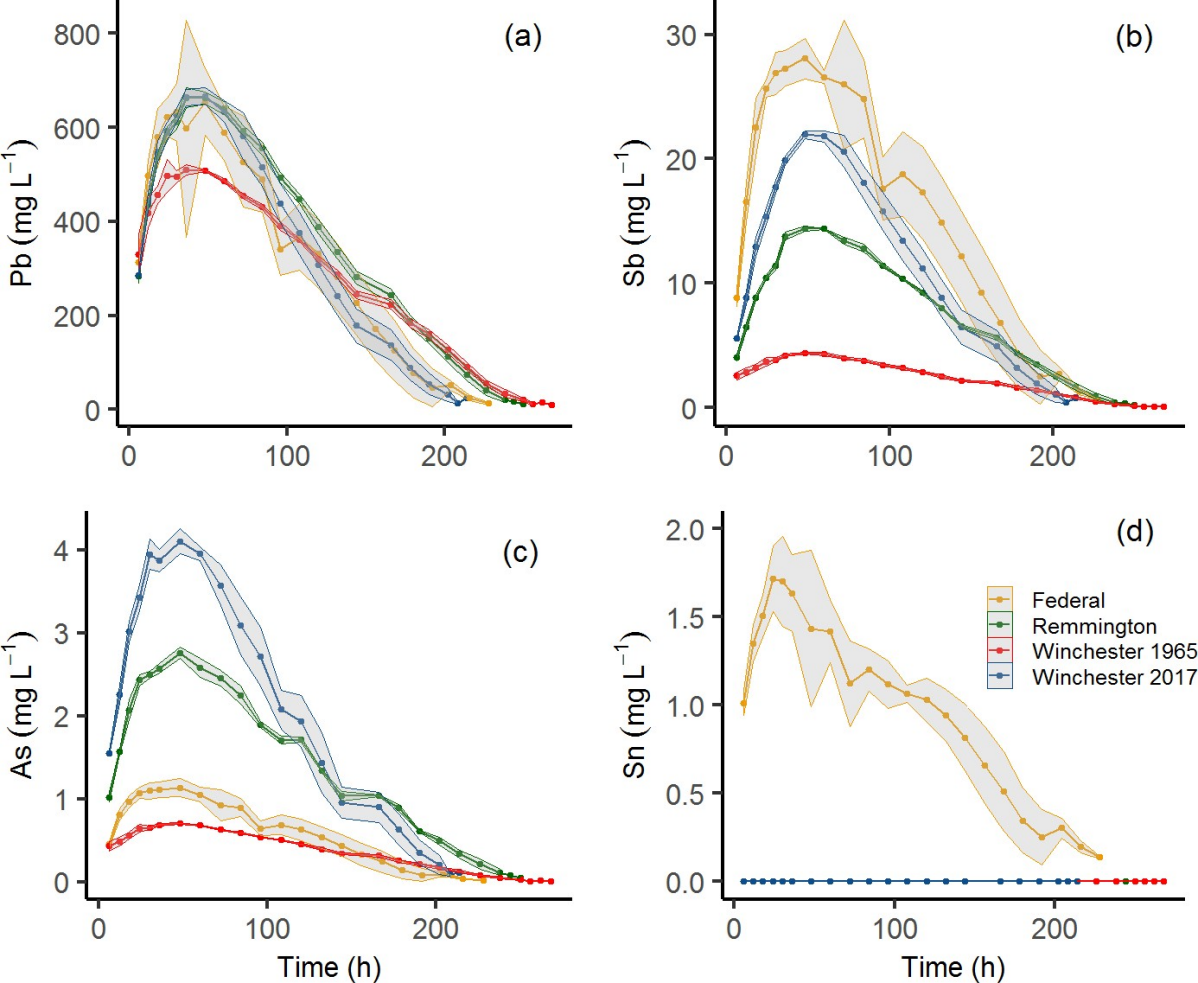
Average Pb, Sb, As, and Sn concentrations for all four shot types. **(a)** Pb, **(b)** Sb, **(c)** As, and **(d)** Sn. Winchester and Remington shot are not shown in **(d)** because Sn was not detected at any time point. Shaded areas represent the standard deviation of the replicate samples (*n* = 6 for Federal shot, *n* = 3 for all other shot types).

Lead concentrations ranged from 9.1 to 667 μg/mL for all brands and time intervals (Fig 4A). Lead concentrations in solution increased for the first 36–48 hours for all types of ammunition and then declined for the remainder of the experiment. Winchester (2017) had the greatest total concentrations of Pb, followed by Remington (2017), Federal (2011), and Winchester (1965). Initial pellet mass and total Pb concentration were linearly and positively correlated (S1 Fig; *P* < 0.01; r^2^ = 0.82).

**Fig 5:**
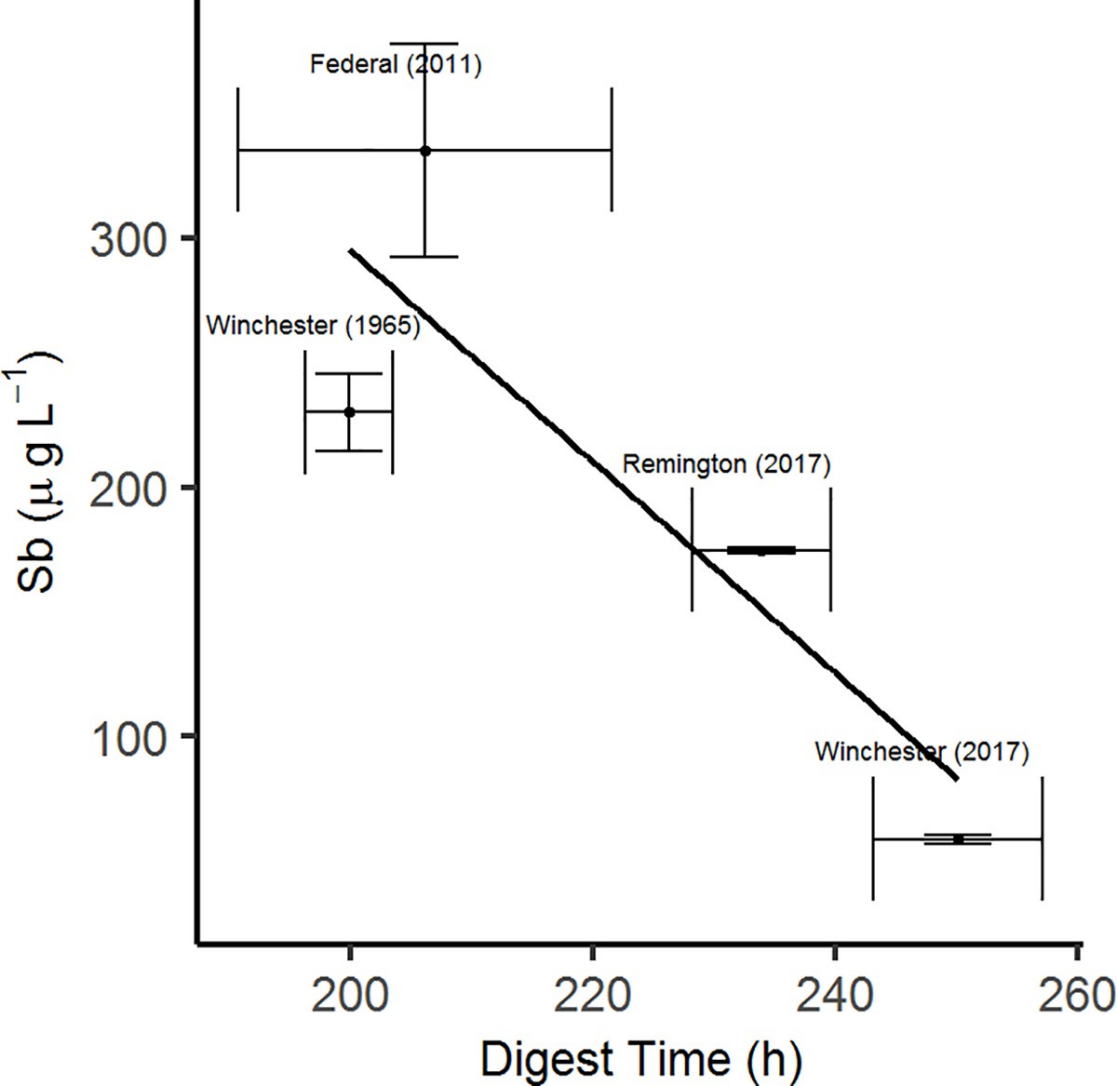
Average Sb concentrations of each Pb shot brand compared to average total digestion time. Among the four shot types tested, there was a general negative correlation (y = -4.2462x + 1144.9; r^2^ = 0.7514).

Antimony concentrations ranged from < DL to 28 μg/mL for all brand-year combinations and time points (Fig 4B), where the lowest concentrations were found at the end of the experiment. Federal (2011) had the greatest concentration of Sb, followed by Winchester (2017), Remington (2017), and Winchester (1965). Total Sb concentrations were three to six times lower in the Winchester (1965) pellets (59 ug/mL) compared to the other brands tested (Fig 5).

Arsenic concentrations ranged from < DL to 4.1 μg/mL for all brands and time points (Fig 4C). Winchester (2017) had the greatest concentrations of As (34 μg/mL), followed by 2017 Remington (26 μg/mL), 2011 Federal (11 μg/mL), and 1965 Winchester (7.5 μg/mL; Table 1). Tin was not detected in the Winchester (1965 or 2017) or Remington (2011) shot; however, concentrations in the Federal brand shot ranged from < DL to 1.7 μg/mL for all time points (Fig 4D). For all metal(loid)s, concentrations increased linearly over the first ~24 hours, then decreased until completion of the experiment. While Pb concentrations increased as the initial pellet mass increased, Sb, Sn and As did not exhibit a strong positive relationship (S1 Fig).

**Table 1:** Total averaged concentrations (μg/mL) and percent concentrations (%conc.) for As, Sn, Sb, and Pb in all shot types.

		As		Sn		Sb		Pb	
	n	Total	%conc.	Total	%conc.	Total	%conc.	Total	%conc.
Winchester (1965)	3	7.5 (0.2)	0.13	< DL^a^	-	46 (1.4)	0.82	5500 (125)	99.05
Federal (2011)	6	11 (1.7)	0.17	17	0.27	270 (40)	4.30	6100 (1000)	95.26
Remington (2017)	3	26 (0.1)	0.39	< DL	-	136 (1.0)	2.04	6500 (190)	97.57
Winchester (2017)	3	34 (2.0)	0.57	< DL	-	176 (10)	2.99	5700 (370	96.44

Standard deviations are shown in parentheses.

^a^DL = detection limit (0.5 μg/mL for Sn)

### Bioaccessible concentrations

**Fig 6:**
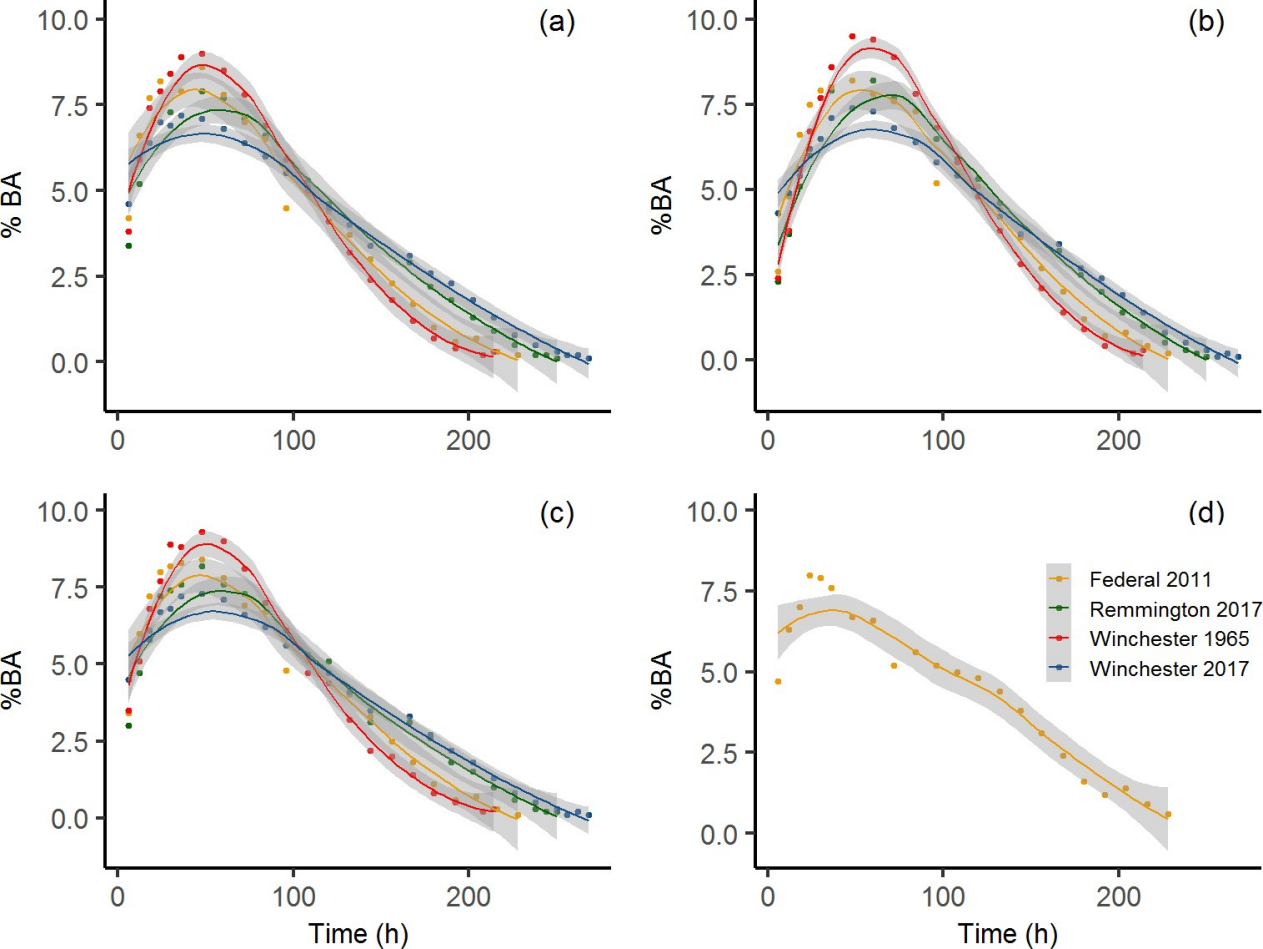
Average percent bioaccessibility of As, Sn, Sb, and Pb for each shot type at all time points analyzed. **(a)** Pb, **(b)** Sb, **(c)** As, and **(d)** Sn. Tin (d) is only included for Federal (2011) as Sn was < DL for all other shot types and bioaccessibility calculations could not be performed. Solid line represents the line of best fit along and shaded areas are the 95% confidence interval (*n* = 6 for Federal shot, *n* = 3 for all other shot types).

Mean concentrations for each brand-year combination ranged from 7.5–34 μg/mL for As, < DL to 17 μg/mL for Sn, 46–270 μg/mL for Sb, and 5500–6500 μg/mL for Pb (Table 1). Total metal(loid) concentrations at each time point were used to determine the %BA, where %BA ranged from 0.1% to 10% for all elements and brands (S1 Table). Maximum Sb %BA was greatest in the 2017 Winchester (9.5%BA) pellets followed by 2017 Remington and 2011 Federal (8.2%BA), and 1965 Winchester (7.4%BA). All elements from all brands followed a similar trend where %BA increased until approximately hour 50, then began to decrease until complete digestion had occurred (S1 Table, Fig 6).

## Discussion

Concentrations and bioaccessibility of As, Sn, and Sb from Pb shot were examined to determine the potential value of using these elements as markers to identify Pb shot exposure in biological samples. This study lends credence to the hypothesis that Sb is a useful marker of Pb shot exposure in biological tissues, particularly when Pb pellets are neither present nor observed in avian tissues. This research demonstrates that Sb is indeed extracted into the gizzard solution, regardless of pellet brand, where pellet residence time as short as 6 hours will provide an exposure pathway for both Pb and Sb absorption into the intestinal tract and distribution throughout the body.

Few studies have examined Pb bioaccessibility directly from Pb shot ingestion in simulated avian environments [47,55,59] and no previous studies report the bioaccessibility of other elements (Sb, Sn, or As) present in Pb shot. Tin was absent in three of the brands analyzed and would not be a reliable indicator of Pb shot exposure. Arsenic can also be eliminated from further consideration, as concentrations and %BA throughout this research did not exceed natural background exposure [60,61]. Finally, preliminary work demonstrated that that Pb and Sb are not present in steel shot (non-toxic alternatives to Pb shot) [31]. Thus, the remainder of the discussion focuses on the feasibility of using Sb as a metallic marker of Pb shot exposure as it was consistently detected throughout this research, regardless of brand.

Previous studies have reported pellet retention times in wild birds range from hours to several weeks, with tremendous variation among species, based upon morphology, food habits, and use of grit [23,56,62]. Based on parameters used in the current study, during the first 6 hours of exposure (ingestion) birds are exposed to ~ 300 μg/mL Pb and ~5 μg/mL Sb and complete pellet dissolution may occur in ~ 9.5 days, which is a similar exposure time in Northern bobwhite as reported by Kerr et al. (2010). Both Pb and Sb concentrations available to birds are expected to increase for 34–48 hours, indicating that the first two days post-consumption are a critical time for exposure to both elements (Fig 4).

Initial pellet mass had a strong impact on total Pb concentrations (S1 Fig) and differences found in Pb concentrations among brands may be solely due to the initial pellet mass. This is clearly observed with the Winchester (2017) pellets as they had greater total Pb concentration than other brand-year combination and the greatest mass. The percentage of Pb and the other trace elements were stable among replicates despite variations in concentrations based on mass, indicating a homogenous mixture of elements for each brand-year combination.

While Pb concentrations were strongly correlated with initial pellet mass, there were weaker correlations between Sb and pellet mass (S1 Fig). This suggests that Sb concentrations are likely affected more by pellet brand than the initial pellet mass, which is not unexpected as different brands typically use different concentrations of Sb in the manufacturing process. Knowing that pellet brand impacts Sb concentrations (and other trace metals) has implications for use in forensic studies on poaching or illegal shot used for hunting. For example, one could link a specific pellet found back to an individual carrying a specific brand of shot using characteristic elements and concentrations found within the pellet as tracers. While the variations in Sb concentrations among brands and manufacture date may be useful in some cases, for Pb source determination it might be cause for concern. For example, it appears that Winchester increased Sb concentration in their manufacturing process between 1965 and 2017 based on this study. While concentrations may have changed from year to year, Sb concentrations were still great enough in both years to cause elevated concentrations in the simulated gizzard juices.

Since Sb is added to Pb shot pellets to increase hardness [28,29], it was originally hypothesized that pellets with a greater concentration of Sb would take longer to digest. However, this hypothesis was not supported (Fig 5). This led to investigating the thermodynamic properties of Pb and Sb, more specifically the standard enthalpy of formation (Gibbs free energy; ΔG_f_) and entropy (S°) for PbCl_2_ and SbCl_3_/SbCl_5,_ as these are the likely compounds of Pb and Sb to form in the gizzard solution. As ΔG_f_ becomes more negative and S° becomes more positive the reaction is more spontaneous and more likely to occur. Based on these values for PbCl_2_, SbCl_3_, and SbCl_5_ (S2 Table) it appears that SbCl_5_ would be most likely to form, followed by SbCl_3_ then PbCl_2_ [63]. Because the formation of the Sb compounds is more likely than the Pb compounds, it can be assumed that greater Sb concentration in a pellet results in faster pellet digestion.

Pellet dissolution rate decreased over time, rather than remaining constant (Fig 2) while cumulative percent mass loss predictably increased (Fig 3B). This decline in dissolution rate was likely due to the increasingly smaller pellet size at each time interval, where less surface area was contacting the sides of the sample tube (mimicking the grinding action occurring in the gizzard) and the gizzard solution itself. Thus, dissolution rate may be greater in a biological setting (i.e., the dissolution rates reported herein are likely more conservative than in a real organismal system) because there would be a more active grinding action occurring from the constriction of the gizzard muscles. In addition, the presence of digestive enzymes (containing sulfur) may aid in the dissolution of Pb shot pellets [15]. The relationship between mass loss and dissolution rate is not linear as expected and suggests that the composition of the pellet plays an important role in determining the dissolution rate.

Total concentrations of Sb extracted into the gizzard solutions were > 46 μg/mL within 11 days in all pellet brands analyzed, where birds would be exposed to continuous concentrations of both Pb and Sb over a short time period from a single pellet. This exposure may lead to absorption into the bloodstream and distribution to organs and tissues. Additionally, this would be amplified by exposure to multiple pellets at the same time or consumption of additional pellets after initial exposure, which is quite likely as pellets tend to be unevenly distributed in the environment. Maximum Sb concentrations were reached within 48 hours in the gizzard solutions, suggesting concentrations of Sb may be greatest in the bloodstream within 48 hours of exposure. Further, even if pellets are excreted within hours or days, exposure to Sb still occurs.

There is little available information on Sb concentrations in avian tissues; thus, further evaluation is needed to determine the practicality of using this metalloid as a metallic marker for Pb shot. For example, dosing studies would be ideal to determine the absorption rate of Sb and which tissues may be best to use to further evaluate the use of Sb as a useful marker for Pb shot exposure. Analyzing for Sb may be a better, and perhaps even cheaper alternative than using Pb isotopic ratios to provide initial information on Pb shot exposure. Isotopic analysis can be expensive and is typically performed after identifying tissues with elevated Pb to narrow the number of samples for analysis. By using Sb as an indicator of Pb shot exposure, both Pb and Sb can be analyzed simultaneously using ICP-MS, potentially decreasing the costs of analyses. Although Pb isotopic analysis would still be useful to confirm Pb shot exposure, its use will continue to be less precise as use of recycled Pb continues to increase–which tends to use Pb from a variety of origins, and would likely mask Pb origin specificity.

### Future considerations

Estimates of exposure and dissolution in this study are likely conservative, since no grinding/pulverizing action (mimicking gizzard mechanical actions) were used. The pellets in this experiment were shaken using an orbital shaker; however, the muscles in the gizzard contract and relax which flattens and distorts pellet shape [64] and would accelerate pellet dissolution in natural gizzards. Pellet retention rates vary drastically among species, and even a short exposure (1–6 hours) to ingested Pb shot can increase Pb and Sb bioaccessibility. Therefore, once a Pb pellet is ingested, absorption of Pb into the blood can be detected almost immediately [43] and would continue until either excreted or completely absorbed in the gizzard. This finding is germane to any future studies of Pb shot exposure using Pb shot presence/absence in gizzards as a proxy for Pb shot exposure. Clearly, absence of pellets in gizzards or GI tracts does not necessarily indicate that birds were not exposed to Pb shot, even as recently as 1–5 days prior to examination.

The physical properties of the pellet affect the bioaccessibility of Pb and the other trace-elements. Weathered pellets (although potentially smaller in size) may better represent the pellets birds are exposed to and have been shown to increase Pb bioaccessibility [44]. When a pellet is exposed to soil, the soil begins to oxidize the pellet creating a layer of Pb (Pb_3_(CO_3_)_2_(OH)_2_, PbCO_3_ and PbSO_4_) [65,66] that is more easily extracted in the digestive system. This would potentially cause a greater level of Pb exposure, or a spike in exposure, upon initial ingestion of the Pb shot pellet, followed by a slower, steady release of Pb for the remaining time the pellet is in the digestive tract. As Pb is assumed to be more bioaccessible from weathered pellets, Sb bioaccessibility may also be increased during this time, further substantiating the notion that estimates of dissolution and bioaccessibility may have been comparatively conservative in this study.

In this study, factors such as pH, food or grit ingestion, soil consumption, and number of pellets consumed were not considered. Martinez-Haro et al. (2009) studied many of the factors to see how they affect Pb absorption. Calcareous grit decreased Pb concentrations found in both the gizzard and intestinal solutions when compared to siliceous grit. The addition of food also decreased Pb concentrations in the simulated gizzard and intestinal solutions. The pH of the gizzard solution can vary between 1.2 and 3.5 [15,55] and is effected by grit type/presence and the presence of food [67,68]. The changing conditions of the gizzard environment can have varied effects on the dissolution of Pb pellets. It is currently unknown how these factors would effect the dissolution of Sb from the Pb shot pellets; however, if these factors physically slow biological pellet degradation, then Sb may follow a similar fate as Pb. Other parameters, such as pellet retention time, intra- and interspecies variation, and environmental conditions can play a role in the bioaccessibility and toxicity of Pb and Sb from Pb shot pellets [48]. Thus this study remains a conservative approach to determining metal bioaccessibility from Pb shot as additional factors known to decrease bioaccessibility were not included.

Another consideration would be any potential exposure to Sb from other non-toxic shot alternatives. This study found no Sb in steel pellets, other non-toxic alternatives include bismuth (Bi) and Tungsten (W). Bismuth is known to contain low levels of Pb (typically < 0.50%) but Sb concentrations are not as well characterized. Only one article was found to report Sb concentrations [28]. They compared information collected on the same shot type from two different entities, one reporting no Sb and the other reporting 0.48% Sb. Most manufactures of Bi shot do not list Sb as a component, which is typically alloyed with greater concentrations of Sn. Thus, Sb is likely just a trace contaminant in the production of Bi shot. Experimental studies including other shot types would be recommended for further study to delineate any potential interferences.

To reliably use Sb as a marker of Pb shot exposure in avian tissues, other sources of Pb exposure must contain low Sb or have low Sb bioaccessibility. Potential environmental sources of Pb in birds include leaded gasoline [69], Pb-arsenate pesticides [70], Pb batteries [71], mining/smelting activies [72], roadside dust [73], and coal combustion [74–76]. Leaded gasoline and Pb-arsenate pesticides can be removed from discussion as they do not contain large concentrations of Sb; thus posing no risk of a false positive. Lead batteries often do contain Sb; however, consumption of large amounts of Pb battery contaminated soils would be unlikely for many species of birds. It has been found that the Pb has a low bioaccessibility (in humans) from soils contaminated from Pb batteries and the overall soil-Pb concentration is less than exposure to Pb shot [77]. Roadside dust is another potential source of Pb and Sb in birds feeding near major roadways; however, Sb concentrations are not great enough to be a substantial source of Sb, especially when compared to the concentrations present Pb ammunition [78–80]. Mining/smelting sites are known to exhibit high soil Pb and Sb concentrations [81, 82]; however, Sb concentrations in various tissues from birds exposed to contaminated mining sites were < 0.5 μg/g [83–85]. Further, Sb bioaccessibility was found to be < 3% from contaminated mining soils [86, 87]. Beyond the Pb source itself, it is also important to account for the amount of contaminated substance consumed. For example, if Pb/Sb concentrations in the focal substrate(s) are high, and are both rarely consumed and poorly absorbed, it may be a largely irrelevant risk. As such, it would be important to perform bioaccessibility studies for both Pb and Sb from other environmentally/ecologically relevant sources to further elucidate the feasibility of using Sb as a marker of Pb shot in biological tissues.

This research provides valuable information into the biological behavior of Pb shot and more specifically the bioaccessibility of Sb and other trace elements. It is recommended that at least seven pellets from each shotgun shell be used in future bioaccessibility studies to capture any pellet composition variation. Further examining the biological degradation of Pb shot pellets using weathered pellets, varied pH, different levels of food, grit, and soil consumption, and a more realistic grinding/pulverizing action will provide a greater understanding of the fate of Pb ammunition.

## Conclusions

There is compelling evidence to support the use of Sb as a metallic marker of Pb shot exposure in biological samples. Even if pellet retention time is on the order of hours, rather than days, detectable concentrations of Pb and Sb are found in the simulated gizzard solution. This study provides evidence that Sb is bioaccessible in the avian digestive system following consumption of Pb shot pellets regardless of the brand and age of the pellets. Adding Sb to analyses when analyzing for Pb and Pb toxicity may provide additional insight into the primary sources of Pb exposure.

## Supporting information

S1 Fig Pb, Sb, Sn and As concentrations for each Pb shot brand compared to initial pellet mass. **(a)** Pb, **(b)** Sb, **(c)** Sn, and **(d)** As; Pb shows a strong positive relationship, while Sb, Sn and As show very weak relationships to the initial pellet mass. Note: due to large variations in concentrations between metals, each is on a different scale. Pb: y = 74.198x + 2023.4; r^2^ = 0.8152; *n* = 15; Sb: y = 5.9381x + 214.58; r^2^ = 0.2687; *n* = 15; Sn: y = -0.2092x + 35.57; r^2^ = 0.1655; *n* = 6; As: y = 0.179x + 9.4094; r^2^ = 0.0153; *n* = 15
(DOCX)

S1 Table Average percent bioaccessibility for As, Sn, Sb, and Pb for each Pb shot brand. Tin is not reported for Winchester (1965, 2017) or Remington (2017) because all concentrations were < DL. Gaps in data are due to the varying digestion times among brands and individual pellets. The number of samples for each brand are as follows: *n* = 3 for Winchester (1965), *n* = 7 for Federal (2011), *n* = 3 for Remington (2017), and *n* = 3 for Winchester (2017).
(DOCX)

S2 Table Standard enthalpy of formation (ΔG_f_) and entropy (S°) values for PbCl_2_, SbCl_3_, and SbCl_5_. Values were obtained from the *Handbook of Inorganic Chemicals*.
(DOCX)
