# Supplementary material for: Bioaccessibility of antimony and other trace elements from lead shot pellets in a simulated avian gizzard environment
Source: PLoS One. 2020 Feb 11;15(2):e0229037. doi: 10.1371/journal.pone.0229037 (PMC7012451; doi:10.1371/journal.pone.0229037)
Supplement: S1 Fig — (a) Pb, (b) Sb, (c) Sn, and (d) As; Pb shows a strong positive relationship, while Sb, Sn and As show very weak relationships to the initial pellet mass. Note: due to large variations in concentrations between metals, each is on a different scale. Pb: y = 74.198x + 2023.4; r2 = 0.8152; n = 15; Sb: y = 5.9381x + 214.58; r2 = 0.2687; n = 15; Sn: y = -0.2092x + 35.57; r2 = 0.1655; n = 6; As: y = 0.179x + 9.4094; r2 = 0.0153; n = 15 (DOCX) [file pone.0229037.s001.docx]

**Bioaccessibility of antimony and other trace elements from lead shot pellets in a simulated avian gizzard environment**

Amanda D. French,^1,#a^* Katherine Shaw,^1^ Melanie Barnes,^2^ Jaclyn E. Cañas-Carrell,^1^ Warren C. Conway,^3^ David M. Klein^1^

^1^ Department of Environmental Toxicology, The Institute of Environmental and Human Health,

Texas Tech University, Lubbock, Texas, United States of America

^2^ Department of Geosciences, Texas Tech University, Lubbock, Texas, United States of America

^3^ Department of Natural Resources Management, Texas Tech University, Lubbock, Texas, United States of America

^#a^Current address: School of Science, University of Waikato, Hamilton, New Zealand

*Corresponding author

E-mail: amanda.french@waikato.ac.nz


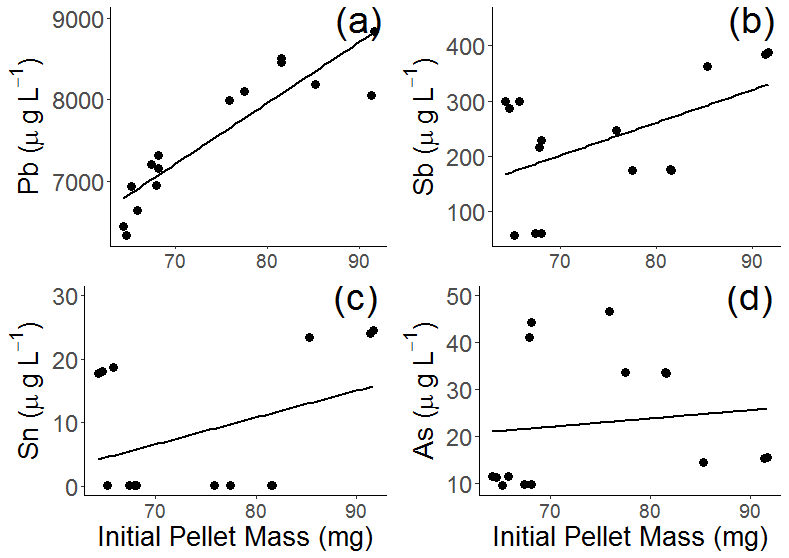


**S1 Fig:** **(a)** Pb, **(b)** Sb, **(c)** Sn, and **(d)** As concentrations (µg/mL) of each pellet analyzed compared to initial pellet mass. Pb shows a strong positive relationship, while Sb, Sn and As show very weak relationships to the initial pellet mass. Note: due to large variations in concentrations between metals, each is on a different scale. Pb: y = 74.198x + 2023.4; r^2^ = 0.8152; *n* = 15; Sb: y = 5.9381x + 214.58; r^2^ = 0.2687; *n* = 15; Sn: y = -0.2092x + 35.57; r^2^ = 0.1655; *n* = 6; As: y = 0.179x + 9.4094; r^2^ = 0.0153; *n* = 15
