# Supplementary material for: Bioaccessibility of antimony and other trace elements from lead shot pellets in a simulated avian gizzard environment
Source: PLoS One. 2020 Feb 11;15(2):e0229037. doi: 10.1371/journal.pone.0229037 (PMC7012451; doi:10.1371/journal.pone.0229037)
Supplement: S1 Table — Tin is not reported for Winchester (1965, 2017) or Remington (2017) because all concentrations were < DL. Gaps in data are due to the varying digestion times among brands and individual pellets. The number of samples for each brand are as follows: n = 3 for Winchester (1965), n = 7 for Federal (2011), n = 3 for Remington (2017), and n = 3 for Winchester (2017). (DOCX) [file pone.0229037.s002.docx]

**Bioaccessibility of antimony and other trace elements from lead shot pellets in a simulated avian gizzard environment**

Amanda D. French,^1,#a^* Katherine Shaw,^1^ Melanie Barnes,^2^ Jaclyn E. Cañas-Carrell,^1^ Warren C. Conway,^3^ David M. Klein^1^

^1^ Department of Environmental Toxicology, The Institute of Environmental and Human Health,

Texas Tech University, Lubbock, Texas, United States of America

^2^ Department of Geosciences, Texas Tech University, Lubbock, Texas, United States of America

^3^ Department of Natural Resources Management, Texas Tech University, Lubbock, Texas, United States of America

^#a^Current address: School of Science, University of Waikato, Hamilton, New Zealand

*Corresponding author

E-mail: [amanda.french@waikato.ac.nz](mailto:amanda.french@waikato.ac.nz)

|  | Winchester (1965) | | | Federal (2011) | | | | Remington (2017) | | | Winchester (2017) | | |
| --- | --- | --- | --- | --- | --- | --- | --- | --- | --- | --- | --- | --- | --- |
| Time (h) | As | Sb | Pb | As | Sn | Sb | Pb | As | Sb | Pb | As | Sb | Pb |
| 6 | 4.5 | 4.3 | 4.6 | 3.4 | 4.7 | 2.6 | 4.2 | 3.0 | 2.3 | 3.4 | 3.5 | 2.4 | 3.8 |
| 12 | 5.1 | 4.9 | 5.9 | 6.0 | 6.3 | 4.8 | 6.6 | 4.7 | 3.7 | 5.2 | 5.1 | 3.8 | 5.9 |
| 18 | 5.8 | 5.4 | 6.4 | 7.2 | 7.0 | 6.6 | 7.7 | 6.1 | 5.1 | 6.4 | 6.8 | 5.6 | 7.4 |
| 24 | 6.7 | 6.2 | 7.0 | 8.0 | 8.0 | 7.5 | 8.2 | 7.2 | 6.0 | 7.0 | 7.7 | 6.7 | 7.9 |
| 30 | 6.8 | 6.5 | 6.9 | 8.2 | 7.9 | 7.9 | 8.4 | 7.4 | 6.5 | 7.3 | 8.9 | 7.7 | 8.4 |
| 36 | 7.2 | 7.1 | 7.2 | 8.3 | 7.6 | 8.0 | 7.9 | 7.6 | 7.9 | 7.9 | 8.8 | 8.6 | 8.9 |
| 48 | 7.3 | 7.4 | 7.1 | 8.4 | 6.7 | 8.2 | 8.6 | 8.2 | 8.2 | 7.9 | 9.3 | 9.5 | 9.0 |
| 60 | 7.1 | 7.3 | 6.8 | 7.8 | 6.6 | 7.8 | 7.8 | 7.6 | 8.2 | 7.7 | 9.0 | 9.4 | 8.5 |
| 72 | 6.6 | 6.8 | 6.4 | 6.9 | 5.2 | 7.6 | 7.0 | 7.3 | 7.7 | 7.1 | 8.1 | 8.9 | 7.8 |
| 84 | 6.2 | 6.4 | 6.0 | 6.7 | 5.6 | 7.3 | 6.5 | 6.7 | 7.3 | 6.6 | 7.0 | 7.8 | 6.9 |
| 96 | 5.6 | 5.8 | 5.5 | 4.8 | 5.2 | 5.2 | 4.5 | 5.6 | 6.5 | 5.9 | 6.1 | 6.8 | 5.9 |
| 108 | 5.3 | 5.4 | 5.1 | 5.1 | 5.0 | 5.5 | 4.9 | 5.1 | 5.9 | 5.3 | 4.7 | 5.8 | 5.0 |
| 120 | 4.7 | 4.8 | 4.5 | 4.7 | 4.8 | 5.1 | 4.4 | 5.1 | 5.3 | 4.6 | 4.4 | 4.8 | 4.1 |
| 132 | 4.1 | 4.2 | 4.0 | 4.0 | 4.4 | 4.4 | 3.7 | 4.0 | 4.6 | 4.0 | 3.2 | 3.8 | 3.2 |
| 144 | 3.5 | 3.7 | 3.4 | 3.3 | 3.8 | 3.6 | 3.0 | 3.1 | 3.7 | 3.4 | 2.2 | 2.8 | 2.4 |
| 156 | 3.3 | 3.4 | 3.1 | 2.5 | 3.1 | 2.7 | 2.3 | 3.1 | 3.2 | 2.9 | 2.0 | 2.1 | 1.8 |
| 168 | 2.7 | 2.7 | 2.6 | 1.8 | 2.4 | 2.0 | 1.7 | 2.6 | 2.5 | 2.2 | 1.4 | 1.4 | 1.2 |
| 180 | 2.2 | 2.4 | 2.3 | 1.1 | 1.6 | 1.2 | 1.0 | 1.8 | 2.0 | 1.8 | 0.8 | 0.9 | 0.7 |
| 192 | 1.8 | 1.9 | 1.8 | 0.6 | 1.2 | 0.7 | 0.6 | 1.5 | 1.4 | 1.3 | 0.5 | 0.4 | 0.4 |
| 204 | 1.3 | 1.4 | 1.3 | 0.7 | 1.4 | 0.8 | 0.7 | 1.0 | 1.0 | 0.9 | - | - | - |
| 208 | - | - | - | - | - | - | - | - | - | - | 0.2 | 0.2 | 0.2 |
| 216 | 0.8 | 0.8 | 0.8 | 0.3 | 0.9 | 0.4 | 0.3 | 0.6 | 0.5 | 0.5 | 0.3 | 0.3 | 0.3 |
| 228 | 0.5 | 0.5 | 0.5 | 0.1 | 0.6 | 0.2 | 0.2 | 0.3 | 0.3 | 0.2 | - | - | - |
| 234 | - | - | - | - | - | - | - | 0.2 | 0.2 | 0.2 | - | - | - |
| 240 | 0.3 | 0.3 | 0.3 | - | - | - | - | 0.2 | 0.1 | 0.1 | - | - | - |
| 246 | 0.1 | 0.1 | 0.2 | - | - | - | - | - | - | - | - | - | - |
| 252 | 0.2 | 0.2 | 0.2 | - | - | - | - | - | - | - | - | - | - |
| 258 | 0.1 | 0.1 | 0.1 | - | - | - | - | - | - | - | - | - | - |

**S1 Table:** Average percent bioaccessibility (%BA) for As, Sn, Sb, and Pb for each brand of Pb shot analyzed. Tin is not reported for Winchester (1965, 2017) or Remington (2017) because all concentrations were < DL. Gaps in data are due to the varying digestion times among brands and individual pellets. The number of samples for each brand are as follows: *n* = 3 for Winchester (1965), *n* = 7 for Federal (2011), *n* = 3 for Remington (2017), and *n* = 3 for Winchester (2017).
