# Supplementary material for: Bioaccessibility of antimony and other trace elements from lead shot pellets in a simulated avian gizzard environment
Source: PLoS One. 2020 Feb 11;15(2):e0229037. doi: 10.1371/journal.pone.0229037 (PMC7012451; doi:10.1371/journal.pone.0229037)
Supplement: S2 Table — Values were obtained from the Handbook of Inorganic Chemicals. (DOCX) [file pone.0229037.s003.docx]

**Bioaccessibility of antimony and other trace elements from lead shot pellets in a simulated avian gizzard environment**

Amanda D. French,^1,#a^* Katherine Shaw,^1^ Melanie Barnes,^2^ Jaclyn E. Cañas-Carrell,^1^ Warren C. Conway,^3^ David M. Klein^1^

^1^ Department of Environmental Toxicology, The Institute of Environmental and Human Health,

Texas Tech University, Lubbock, Texas, United States of America

^2^ Department of Geosciences, Texas Tech University, Lubbock, Texas, United States of America

^3^ Department of Natural Resources Management, Texas Tech University, Lubbock, Texas, United States of America

^#a^Current address: School of Science, University of Waikato, Hamilton, New Zealand

*Corresponding author

E-mail: [amanda.french@waikato.ac.nz](mailto:amanda.french@waikato.ac.nz)

| Compound | ΔG_f_ (kcal/mol) | S° (cal/degree mol) |
| --- | --- | --- |
| PbCl_2_ | -75.98 | 32.50 |
| SbCl_3_ | -77.4 | 44.0 |
| SbCl_5_ | -83.7 | 72 |

**S2 Table:** Standard enthalpy of formation (ΔG_f_) and entropy (S°) values for PbCl_2_, SbCl_3_, and SbCl_5_. Values were obtained from the *Handbook of Inorganic Chemicals*.
